# The psychometric properties of the Quantitative-Checklist for Autism in Toddlers (Q-CHAT) as a measure of autistic traits in a community sample of Singaporean infants and toddlers

**DOI:** 10.1186/s13229-015-0032-1

**Published:** 2015-06-21

**Authors:** I. Magiati, D. A. Goh, S. J. Lim, D. Z. Q. Gan, J. C. L. Leong, C. Allison, S. Baron-Cohen, A. Rifkin-Graboi, B F P. Broekman, S-M. Saw, Y-S. Chong, K. Kwek, P. D. Gluckman, S. B. Lim, M. J. Meaney, on behalf of the GUSTO working group

**Affiliations:** Department of Psychology, National University of Singapore, #02-24, Block AS4, 9 Arts Link, 117570 Singapore, Singapore; Singapore Institute for Clinical Sciences, Agency for Science, Technology and Research (A* STAR), Singapore, Singapore; Ludmer Centre for Neuroinformatics and Mental Health, McGill University, Verdun, Canada; Department of Psychiatry, Faculty of Medicine, McGill University, Montreal, Canada; Department of Psychological Medicine, Yong Loo Lin School of Medicine, National University of Singapore, National University Health System, Singapore, Singapore; Autism Research Centre, Department of Psychiatry, University of Cambridge, Cambridge, UK; Department of Obstetrics and Gynaecology, Yong Loo Lin School of Medicine, National University of Singapore, National University Health System, Singapore, Singapore; Department of Maternal Fetal Medicine, KK Women’s and Children’s Hospital, Singapore, Singapore; Liggins Institute, University of Auckland, Auckland, New Zealand; Saw Swee Hock School of Public Health, National University of Singapore, National University Health System, Singapore, Singapore; Department of Child Development, KK Women’s and Children’s Hospital, Singapore, Singapore

**Keywords:** Autism spectrum disorder, Autistic traits, Dimensional, Assessment, Measure, Quantitative, Checklist, Toddlers, Factor structure, Psychometric properties

## Abstract

**Background:**

There is growing research evidence that subclinical autistic traits are elevated in relatives of individuals with autism spectrum disorder (ASD), continuously distributed in the general population and likely to share common etiology with ASD. A number of measures have been developed to assess autistic traits quantitatively in unselected samples. So far, the Quantitative-Checklist for Autism in Toddlers (Q-CHAT) is one of very few measures developed for use with toddlers as young as 18 months, but little is known about its measurement properties and factor structure.

**Methods:**

The present study examined internal consistency, factor structure, test-retest stability, and convergent validity of the Q-CHAT in a sample of toddlers in Singapore whose caregivers completed the Q-CHAT at 18 (*n* = 368) and 24 months (*n* = 396).

**Results:**

Three factors were derived accounting for 38.1 % of the variance: social/communication traits, non-social/behavioral traits, and a speech/language factor. Internal consistency was suboptimal for the total and speech/language scores, but acceptable for the social/communication and non-social/behavioral factor scores. Scores were generally stable between 18 and 24 months. Convergent validity was found with the Pervasive Developmental Disorders subscale of the Child Behavior Checklist (CBCL) completed by caregivers when their children were 24 months. Q-CHAT total scores in this sample were higher than those reported in other unselected samples from the UK.

**Conclusions:**

The Q-CHAT was found to have a three-factor structure, acceptable internal consistency for its two main factor scores (social/communication and non-social/behavioral), normally distributed scores in an unselected sample, and similar structure and measurement properties as those reported in other published studies. Findings are discussed in relation to existing literature and future directions for the validation of the Q-CHAT.

## Background

Autism spectrum disorder (ASD) is a group of complex, pervasive, heterogeneous neurodevelopmental conditions characterized by impairments in social communication and interaction and by restricted and circumscribed behaviors and interests [1]. In contrast to the earlier edition of the Diagnostic and Statistical Manual of Mental Disorders (DSM-IV-TR), which differentiated between different subgroups of ASD, a major revision in the DSM-5 is the removal of the categorically defined ASD subgroups and the creation of a uni-dimensional diagnostic category of ASD [2]. This shift in the way we conceptualize ASD stems in part from increased recognition of the limitations of the categorical approach. In clinical practice, while diagnostic stability for the broader diagnosis of ASD is high, ASD subgroup diagnostic classification is often difficult and unreliable [2, 3]. An inability to identify discrete ASD phenotypes [4] suggests that exploring quantitative, rather than qualitative, differences in individuals with ASD and in those in the general population is likely to be a reliable and valid approach to studying autistic symptoms and traits in clinical and unselected populations [5].

Traits are “habitual patterns of behaviour, thought, and emotion, which are stable over time and exist in all individuals to a varying degree” ([6], p. 66). Family studies have provided consistent evidence of a broader autism phenotype (BAP—elevated, but subclinical, levels of autistic traits) among relatives of individuals with ASD [7–11]. There is also growing research evidence that subclinical social, communication, and behavioral autistic traits are present to varying degrees in the general population. A number of population studies have demonstrated the heritability and continuous distribution of autistic traits in community samples of children, adolescents, and adults [12–16]. Studies in different age groups and countries using different quantitative caregiver- or self-report measures of autistic traits have consistently reported that individuals with ASD score significantly higher compared to individuals without ASD with large effect size differences, suggesting that clinical scores fall in the higher ends of the continuum of autistic traits [17–21]. Finally, twin and other studies investigating autistic traits in the general population and in those with ASD have shown that the etiology of autistic traits is likely to be more similar than different to that of ASD (see [22] for a review). For these reasons, it has been proposed that measuring, quantifying, and understanding patterns, trajectories, and correlates of autistic traits in unselected samples can potentially further our understanding of the continuum of autistic traits and of ASD [6].

A number of screening and diagnostic tools that attempt to quantify autistic traits and symptoms as a continuous, rather than categorical, variable have been developed. These include the Autism Spectrum Quotient (AQ; [23]) and more recently the Social Responsiveness Scale-2 (SRS-2) [24] for adults; the Autism Spectrum Screening Questionnaire (ASSQ) [25], Childhood Autism Spectrum Test (CAST) [26], Children’s Social Behaviour Questionnaire [27], and SRS [28] for children and adolescents; and the Quantitative Checklist for Autism in Toddlers (Q-CHAT) [17], and Social Responsiveness Scale-2 Preschool form (SRS-2) [24] for toddlers.

The Q-CHAT is a 25-item caregiver-report screening measure of autistic traits for toddlers aged 18–24 months measuring developmentally relevant traits and behaviors relating to autism, including joint attention, pretend play, social communication, stereotyped behaviors, sensory interests, and language development. These domains were initially identified using the ICD-10 and DSM-IV-TR core or common associated features of ASD. Adopting a dimensional-quantitative approach, each Q-CHAT item is scored on a 5-point scale which allows respondents to report the relative frequency, typicality, or severity of observed autistic traits rather than their absolute presence or absence, as in the dichotomous yes/no ratings of the original Checklist for Autism in Toddlers (CHAT) [29], from which the Q-CHAT was developed.

In the first study to employ and report on the Q-CHAT involving caregivers of 754 toddlers aged 18–24 months in the UK, the Q-CHAT total scores were normally distributed, had an internal consistency *α* value of .67, and excellent test-retest reliability after 1 month (*r* = .82) [17]. In the same study by Allison and colleagues [17], significantly higher Q-CHAT total scores (*M* = 51.8, SD = 14.3) were reported for 41 children with established clinical diagnoses of ASD aged 3 years and below as compared to the unselected sample (*M* = 26.7, SD = 7.8), providing preliminary evidence of discriminant validity. Sensitivity, specificity, cutoff values, or the Q-CHAT’s factor structure were not examined. Using data from the same unselected sample as [17], Allison et al. [18] then identified the 10 Q-CHAT items that best discriminated between toddlers with and without ASD. The Q-CHAT-10 had excellent internal consistency (*α* = .88) and was highly correlated with the original 25-item Q-CHAT (*r* = .79).

In another study by Wong and colleagues, the Q-CHAT was used to measure autistic traits quantitatively in a sample of 141 toddlers from the UK who were born very preterm (<30 weeks of gestation) [30]. Q-CHAT total scores were also normally distributed, but significantly higher (*M* = 33.7, SD = 8.3) compared to those from Allison et al.’s unselected sample [17] (calculated effect size *d* = .87). In this study [30], the Q-CHAT items were conceptually classified into four categories: social-relatedness, restricted, repetitive, stereotyped behaviors, communication, and sensory abnormalities, but no factor analysis or calculated factor/category scores were reported. The researchers then compared their participants’ mean item scores against those reported by Allison et al. [17] and found that caregivers of very preterm toddlers reported significantly higher scores in 17 Q-CHAT items, with more differences in items relating to stereotyped behaviors, communication, and sensory abnormalities.

Another two studies [31, 32] have employed the Q-CHAT as a measure of autistic traits, but no further examination or reporting of its psychometric properties has been carried out. No published study has yet examined the factor structure of the Q-CHAT. Allison and colleagues presented a preliminary factor analysis based on their unselected community toddler sample at an international conference [33]. In this preliminary unpublished analysis, 18 of the 25 Q-CHAT items loaded > .35 onto one of the three main factors derived: (i) social interaction (6 items), (ii) communication/language (3 items), and (iii) repetitive and stereotyped behaviors (8 items).

To summarize, the Q-CHAT has so far only been employed in five studies in the UK [17, 18, 30–32] and very limited evidence exists regarding its measurement properties, despite this being, to our knowledge, the only available measure for quantitatively assessing autistic traits in children as young as 18 months.

The present study, thus, aimed to:

(i) quantify, measure, and report the distribution of autistic traits in a sample of Asian toddlers using the Q-CHAT at 18 and 24 months;
(ii) examine the factor structure of the Q-CHAT;
(iii) report the internal consistency of the Q-CHAT total and derived factor scores;
(iv) examine stability and change of Q-CHAT total, factor, and item scores from 18 to 24 months; and
(v) examine the Q-CHAT’s convergent validity with the Pervasive Developmental Problems subscale of the Child Behavior Checklist (CBCL 1.5-5) [30].

## Methods

### Participants

Participants were from a sample of mothers and children who were recruited in an ongoing prospective longitudinal birth cohort study in Singapore (GUSTO—Growing Up in Singapore Towards Healthy Outcomes^1^; see [34] for more details). Participating infants were born between November 2009 and May 2011. Mother-child pairs were followed up at regular intervals from 12 weeks of gestation to 41 months of age, with planned follow-ups taking place currently until at least age 9.

From the “main GUSTO cohort” (*n* = 1162), a subsample of participants were invited for more detailed follow-up neurocognitive phenotyping and neuropsychological assessments at regular intervals, the “GUSTO Neurodevelopmental Cohort”^2^. At 18 months, 431 toddlers and their caregivers participated in the scheduled neurocognitive follow-up; at 24 months, 514 caregiver-toddler pairs participated. The caregivers were invited to complete the Q-CHAT among other measures. Response rates for the Q-CHAT were 85.4 % at 18 months (*n* = 368; 54.2 % males) and 77 % at 24 months (*n* = 396; 51.4 % males), while 294 participants (52.5 % males) had Q-CHAT data at both time points*.* At 24 months, 359 (90.7 %) respondents were mothers, 16 (4.0 %) were fathers, 8 (2.2 %) were grandmothers, aunts, domestic helpers, or more than one respondent, and 13 (3.1 %) did not indicate their relationship to the child^3^.

Q-CHAT completers at 18 and 24 months were compared to the active GUSTO cohort on various demographic variables to examine the representativeness of the present sample (see Table 1). Q-CHAT participants at 18 and at 24 months did not differ significantly from the active GUSTO cohort on maternal age, maternal education, or household monthly income (all *p* > .05; Table 1). However, the 18-month Q-CHAT sample had somewhat more Malay, fewer Chinese, and fewer Indian children than the active GUSTO cohort. The 24-month Q-CHAT sample included more Malay, more Chinese, and fewer Indian children than the main GUSTO cohort (Table 1). Nevertheless, the sizes of these differences were small, indicating that overall, the Q-CHAT sample was reasonably representative of and similar to the active GUSTO cohort in most demographic variables.

**Table 1:** Comparison of key demographics between the Q-CHAT sample at 18 and 24 months and the full active GUSTO cohort

	18 months	24 months	Active GUSTO cohort *n* = 1089	Statistics (differences between 18 M Q-CHAT and active GUSTO)	Statistics (differences between 24 M Q-CHAT and active GUSTO)
	Q-CHAT	Q-CHAT			
	*n* = 368	*n* = 396			
Maternal age, years (SD)	30.3 (5.2)	30.5 (5.2)	30.8 (5.1)	*t*(367) = −1.75, *p* = .08, *d* = .10	*t*(395) = −1.29, *p* = .20, *d* = .06
Ethnicity					
Chinese	195 (53.0 %)	232 (58.6 %)	616 (56.6 %)	*χ* ^2^(2) = 10.64, *p* = .01, *w* = .17	*χ* ^2^(2) = 7.35, *p* = .03, *w* = .14
Malay	119 (32.3 %)	113 (28.5 %)	277 (25.4 %)		
Indian	53 (14.4 %)	51 (12.9 %)	196 (18.0 %)		
Highest maternal education level					
None/primary	14 (3.8 %)	16 (4.0 %)	42 (3.9 %)	*χ* ^2^(2) = 0.31, *p* = .86, *w* = .03	*χ* ^2^(2) = 0.15, *p* = .93, *w* = .02
Secondary/technical education	126 (34.2 %)	139 (35.1 %)	392 (36.0 %)		
GCE ‘A’ levels/polytechnic/university/others	223 (60.6 %)	240 (60.6 %)	655 (60.1 %)		
Household monthly income, SGD^a^					
0–999	9 (2.4 %)	8 (2.0 %)	22 (2.2 %)	*χ* ^2^(4) = 0.76, *p* = .94, *w* = .05	*χ* ^2^(4) = 0.11, *p* =1.00, *w* = .02
1000–1999	44 (12.0 %)	49 (12.4 %)	136 (13.3 %)		
2000–3999	106 (28.8 %)	113 (28.5 %)	304 (29.8 %)		
4000–5999	85 (23.1 %)	92 (23.2 %)	254 (24.9 %)		
≥6000	102 (27.7 %)	114 (28.8 %)	303 (29.7 %)		

^a^According to the Department of Statistics Singapore, in 2013, median household income for Singaporean/Permanent Resident families with at least one working member was SGD7870

### Measures

#### Autistic traits

The Q-CHAT [17] is a 25-item caregiver-report screening measure of autistic traits for toddlers aged 18–24 months. Items are rated on a 5-point Likert scale (0–4) with higher ratings indicating more autistic traits. Thirteen items are reverse scored. Examples of items include “Does your child place your hand on an object when s/he wants you to use it?,” “Does your child look at you when you call his/her name?,” “Does your child do the same thing over and over again?,” and “Does your child echo things s/he hears (e.g. lines from songs or movies, things that you say)?.” Individual item scores are summed up to obtain a Q-CHAT total score, ranging from 0 to 100. More information about the Q-CHAT’s psychometric properties can be found in the “Background” section.

#### Children’s emotional and behavioral problems

The Child Behaviour Checklist 1.5-5 years (CBCL 1.5-5) [35] is a widely used 99-item caregiver report which screens for a range of emotional and behavioral problems in young children aged 1½–5 years old. Rated by caregivers on a 0–2 scale, a Total Problems CBCL raw score (0–198), seven syndrome scale scores, and two aggregate factors of Internalizing and Externalizing Problems are calculated [35]. Five DSM-oriented scales have also been identified, including a Pervasive Developmental Problems 13-item subscale [35, 36].

The CBCL 1.5-5 has been used in multicultural comparisons across 24 societies, including Singapore [37]. In the present study, the CBCL 1.5-5 was completed by caregivers when their children were 24 months old. The Q-CHAT’s concurrent and convergent validity was examined by correlating it with the CBCL Pervasive Developmental Problems (PDD), Withdrawn, and Internalizing Problems scales, as these have been found to discriminate between toddlers with ASD and those with or without other conditions [38–40]. The CBCL PPD subscale is not a measure of autistic traits, but assesses autism-related behavioral problems, and thus, we expected that the Q-CHAT would be positively, but moderately, correlated with this subscale.

### Procedure

Ethical approval was granted by the National Health Care Group Domain Specific Review Board and the Sing Health Centralized IRB and approved by the National University of Singapore IRB. All participating caregivers gave informed written consent before their participation. There was no obligation to take part in the study, and participants could withdraw at any time point without their standard medical care being affected in any way. Each family was reimbursed for every follow-up clinic or home visit and for the completion of caregiver-completed questionnaires.

All biological, genetic, neuropsychological, and behavioral data collected for the GUSTO study at all time points, including the measures used in the present study, were collected by a team of trained researchers and research assistants, including undergraduate and post-graduate psychology students for the neuropsychological and behavioral measures, under supervision by post-doctoral research fellows and senior research staff.

### Missing data and statistical analyses

As per Allison et al. [17], incomplete or ambiguously answered Q-CHAT items were conservatively scored ‘0’. If seven or more Q-CHAT items were missing, then the checklist was excluded from analyses (*n* = 1, 0.3 % at 18; and *n* = 3, 0.9 % at 24 months). For the CBCL at 24 months, questionnaires with more than eight missing items were excluded (*n* = 10, 2.5 %; [41]. For those with eight or fewer missing items, a conservative score of ‘0’ was given to any missing items [42].

All statistical analyses were conducted using the Statistical Package for Social Sciences (SPSS), Version 21.0. Descriptive statistics were first calculated, and data were explored for normality and outliers. An Exploratory Factor Analysis (EFA) using Principal Axis Factoring was then conducted to investigate the factor structure of the Q-CHAT at 18 months. EFA was selected, as it assumes that items on the Q-CHAT cluster according to underlying constructs. Oblique direct oblimin rotation was performed, as psychological constructs/factors are often correlated [43].

Cronbach’s alphas were then calculated to examine the Q-CHAT’s total and derived factor items’ internal consistency at both time points. One sample *t* tests compared total Q-CHAT scores in this Asian sample against Q-CHAT scores obtained in published literature with samples from the UK. Item-total correlations were also examined using Spearman’s *rho* non-parametric analyses. The relationship between 18- and 24-month Q-CHAT scores was examined with Pearson’s *r* correlation analyses for total and factor scores and Spearman’s *rho* for the Q-CHAT items, while paired *t* tests or non-parametric Wilcoxon’s tests examined differences between Q-CHAT total, factor, and item scores between 18 and 24 months. Finally, convergent validity between the Q-CHAT and the CBCL 1.5-5’s Total Problems, Internalizing, Withdrawn, and DSM-oriented PDD problem scales was examined using either Pearson’s *r* or Spearman’s *rho* correlations, depending on the scores’ distribution. Because of the number of statistical tests carried out, the magnitude of effect sizes was carefully considered in the interpretation of the findings.

## Results

### Descriptive statistics

**Fig. 1:**
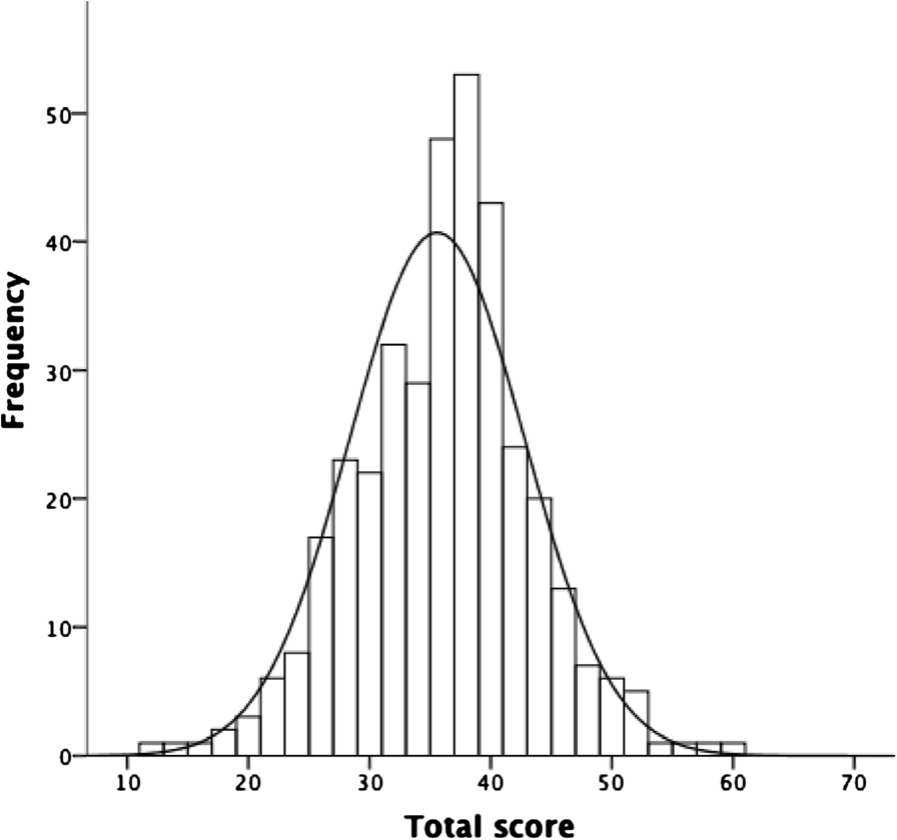
Distribution of 18-month Q-CHAT total scores (*n* = 368)

**Fig. 2:**
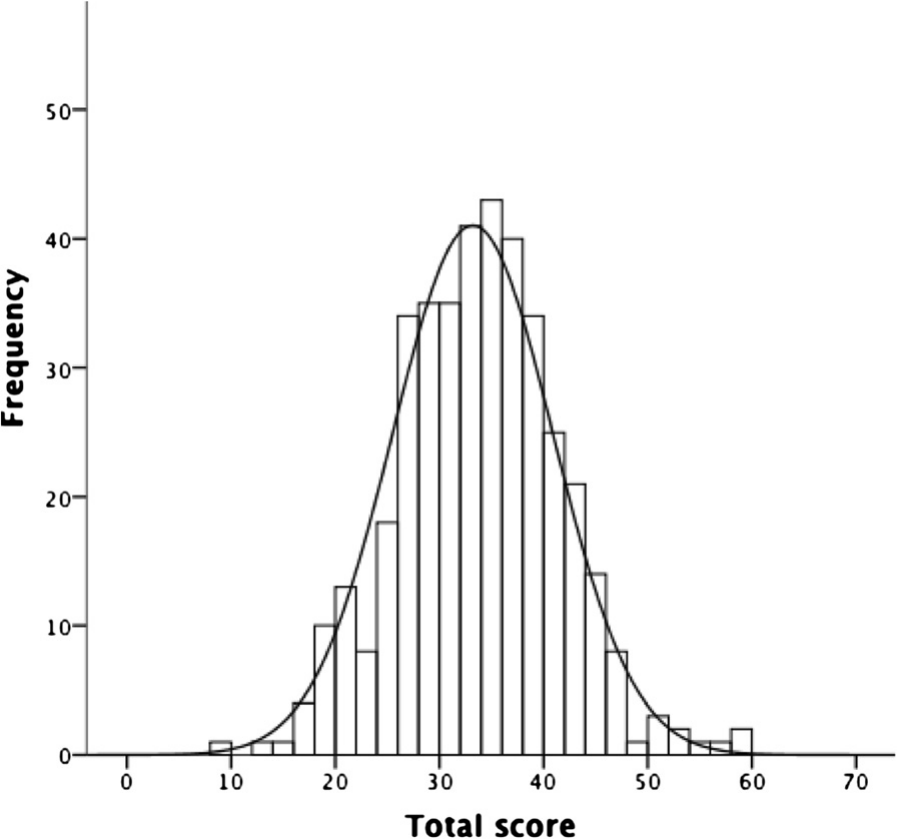
Distribution of 24-month Q-CHAT total scores (*n* = 396)

Q-CHAT scores at 18 and 24 months were normally distributed (see Figs. 1 and 2). Table 2 presents Q-CHAT descriptive statistics at 18 and 24 months.

**Table 2:** Total and Factor Q-CHAT raw scores at 18 and 24 months

	18 months (*n* = 368)		24 months (*n* = 396)		Statistics	
	*M* (SD)	Range	*M* (SD)	Range	Difference between Q-CHAT scores at 18 and 24 months	Relationship between Q-CHAT scores at 18 and 24 months (*r*)
Q-CHAT 25 (total)	35.57 (7.21)	12–59	33.20 (7.70)	9–59	*t*(293) = 6.01, *p* < .0001, *d* = .32	.64***
Q-CHAT social-communicative ALTs (factor 1; 10 items)	9.94 (4.18)	2–27	10.62 (4.51)	2–33	*t*(293) = −3.17, *p* = .002, *d* = .16	.64***
Q-CHAT non-social/behavioral ALTs (factor 2; 8 items)	13.05 (5.11)	0–31	10.80 (5.14)	0–26	*t*(293) = 8.62, *p* < .0001, *d* = .45	.60***
Q-CHAT speech/language (factor 3; 4 items)	8.43 (2.02)	3–16	7.28 (1.73)	1–12	*t*(293) = 8.94, *p* < .0001, *d* = .56	.43***

***p < .001

### Comparison of Q-CHAT scores with those in existing literature

Mean Q-CHAT total scores at 18 (*M* = 35.6, SD = 7.2) and 24 months (*M* = 33.2, SD = 7.7) in this sample were significantly higher (i.e., more autistic traits) than those in Allison and colleagues’ [17] unselected sample of 754 UK toddlers with a mean age of 21.2 months (*M* = 26.7, SD = 7.8; one sample *t*(367) = 23.6, *p* < .001 at 18 months; *t*(395) = 16.8, *p* < .001 at 24 months) with large calculated effect size differences (*d* = 1.18 and .84, respectively). Similarly, our scores were significantly higher than those of Auyeung et al. [31] from 129 19-month-old toddlers (*M* = 26.55, SD = 7.08; *t*(367) = 24.0, *p* < .001 at 18 months; *t*(395) = 17.18, *p* < .001 at 24 months), with large calculated effect size differences (*d* = 1.26 and .90, respectively). Differences were small when our sample’s total scores at 18 and 24 months were compared to those reported by Wong et al. [30] for 2-year-old premature children in the UK (*M* = 33.7, SD = 8.3; *t*(367) = 4.98 *p* < .001 at 18 months; *t*(395) = −1.3, *p* = .19 at 24 months; calculated effect sizes were small, *d* = .24 and − .06 for the two time points, respectively).

### Q-CHAT item distribution and item-total correlations

The distribution of the 25 Q-CHAT item ratings at both time points can be found in Table 3, while mean item scores are presented in Table 4. *Use of caregiver’s hand as a tool* (item 12) and *Echolalia* (item 18) were the two most highly endorsed behaviors at 18 and 24 months, respectively. *Unusual gestures* (item 19) was the least frequently reported behavior at both time points.

**Table 3:** Distribution (%) of Q-CHAT’s item ratings at 18 (18 M) and 24 months (24 M)

	Q-CHAT item ratings (%)									
	0		1		2		3		4	
Q-CHAT items	18 M	24 M	18 M	24 M	18 M	24 M	18 M	24 M	18 M	24 M
1. Looks when name called	60.3	60.4	30.4	31.6	7.9	6.8	1.4	1.3	0.0	0.0
2. Eye contact	46.7	52.3	45.7	40.2	6.8	6.8	0.8	0.5	0.0	0.3
3. Lines objects up^a^	10.1	6.1	32.6	17.4	37.8	45.5	14.7	19.9	4.9	11.1
4. Understand child’s speech	9.0	10.9	20.4	35.1	39.9	41.2	16.6	9.8	14.1	3.0
5. Proto-imperative pointing	76.4	64.1	17.7	23.5	4.6	9.6	0.8	1.8	0.5	1.0
6. Proto-declarative pointing	59.5	54.5	25.8	29.0	10.6	12.4	2.2	2.8	1.9	1.3
7. Interest maintained by spinning object^a^	19.8	21.2	46.7	47.5	23.1	19.7	7.9	7.6	2.4	4.0
8. Number of words^a^	1.4	10.6	3.8	11.1	31.5	49.7	48.6	23.7	14.7	4.8
9. Pretend play	40.8	24.5	35.6	35.4	18.8	23.0	1.9	10.1	3.0	7.1
10. Follows a look	41.3	33.6	37.5	41.9	16.0	18.9	4.6	3.3	0.5	2.3
11. Sniffs/licks unusual objects^a^	17.4	25.8	19.3	35.1	21.2	20.7	27.2	2.6	14.9	5.8
12. Uses hand as tool^a^	6.5	6.3	4.3	7.8	14.9	19.2	29.1	32.6	45.1	34.1
13. Walks on tiptoes^a^	27.2	24.5	19.8	28.3	37.2	37.6	9.5	5.8	6.3	3.8
14. Adapts to changes in routine	19.8	17.7	67.1	68.2	11.7	11.1	1.1	1.5	0.3	1.5
15. Offers comfort	20.9	23.5	29.1	28.8	29.6	31.3	13.6	11.4	6.8	5.1
16. Does same thing over and over again^a^	5.2	12.1	6.8	14.1	16.8	23.2	32.1	30.3	39.1	20.2
17. Typicality of first words	37.0	43.4	44.6	47.0	8.4	6.1	3.0	1.5	7.1	2.0
18. Echolalia^a^	9.0	6.3	9.0	5.1	16.6	15.9	37.5	32.1	28.0	40.7
19. Gestures	75.0	68.2	19.6	25.5	4.6	5.6	0.8	0.3	0.0	0.5
20. Unusual finger movements^a^	38.0	44.2	14.4	18.9	17.4	16.9	20.7	13.1	9.5	6.8
21. Checks reaction	25.5	22.7	38.0	33.6	24.7	33.3	10.3	7.8	1.4	2.5
22. Maintenance of interest^a^	30.4	22.2	36.4	36.6	22.8	30.3	7.1	7.6	3.3	3.3
23. Twiddles objects repetitively^a^	12.2	23.7	16.0	28.0	27.2	24.7	34.5	17.9	10.1	5.6
24. Oversensitive to noise^a^	17.1	22.7	42.4	39.6	25.0	27.0	10.3	7.3	5.2	3.3
25. Stares at nothing with no purpose^a^	64.9	67.9	17.4	19.2	12.5	9.1	3.8	3.0	1.4	0.8

^a^Reverse-scored items

**Table 4:** Q-CHAT item descriptives at 18 (*n* = 368) and 24 months (*n* = 396) and test-retest reliability and differences between mean item scores at 18 and 24 months (*n* = 294)

	18 M	24 M	p***	Difference	Effect size of difference
	Mean (SD)	Mean (SD)		*z*	*r*
1. Looks when name called	0.50 (0.7)	0.49 (0.7)	.49	−.37^b^	.02
2. Eye contact	0.62 (0.7)	0.56 (0.7)	.46	−1.43^b^	.08
3. Lines objects up^a^	1.72 (1.0)	2.13 (1.0)	.38	−5.57***^c^	.38
4. Understand child’s speech	2.07 (1.1)	1.59 (0.9)	.45	−5.88***^b^	.34
5. Protoimperative pointing	0.32 (0.7)	0.52 (0.8)	.27	−3.92***^c^	.23
6. Protodeclarative pointing	0.61 (0.9)	0.67 (0.9)	.40	−1.08^c^	.06
7. Interest maintained by spinning object^a^	1.26 (1.0)	1.26 (1.0)	.42	−.30^b^	.02
8. Number of words^a^	2.71 (0.8)	2.01 (1.0)	.57	−10.69***^b^	.62
9. Pretend play	0.91 (1.0)	1.4 (1.2)	.37	−6.59***^c^	.38
10. Follows a look	0.86 (0.9)	0.99 (0.9)	.43	−2.66**^c^	.15
11. Sniffs/licks unusual objects^a^	2.03 (1.3)	1.38 (1.2)	.32	−7.19***^b^	.42
12. Uses hand as tool^a^	3.02 (1.2)	2.8 (1.2)	.28	−2.11*^b^	.12
13. Walks on tiptoes^a^	1.48 (1.2)	1.36 (1.0)	.51	−1.36^b^	.08
14. Adapts to changes in routine	0.95 (0.6)	1.01 (0.7)	.19	−1.64^c^	.10
15. Offers comfort	1.56 (1.2)	1.46 (1.1)	.57	−2.29*^b^	.13
16. Does same thing over and over again^a^	2.93 (1.1)	2.32 (1.3)	.44	−7.21***^b^	.42
17. Typicality of first words	0.99 (1.1)	0.72 (0.8)	.27	−3.76***^b^	.22
18. Echolalia^a^	2.67 (1.2)	2.96 (1.2)	.38	−4.24***^c^	.25
19. Gestures	0.31 (0.6)	0.39 (0.7)	.36	−1.33^c^	.08
20. Unusual finger movements^a^	1.49 (1.4)	1.19 (1.3)	.47	−3.30**^b^	.19
21. Checks reaction	1.24 (1.0)	1.34 (1.0)	.37	−1.45^c^	.08
22. Maintenance of interest^a^	1.16 (1.0)	1.33 (1.0)	.42	−1.91^c^	.11
23. Twiddles objects repetitively^a^	2.14 (1.2)	1.54 (1.2)	.39	−7.59***^b^	.44
24. Oversensitive to noise^a^	1.44 (1.1)	1.29 (1.0)	.33	−1.66^b^	.10
25. Stares at nothing with no purpose^a^	0.59 (0.9)	0.49 (0.84)	.35	−1.17^b^	.07

*p < .05, **p < .01, ***p < .001 (all *ρ* correlations have *p* < .001 in this table)

^a^Reverse-scored items

^b^Wilcoxon signed-rank test *z*-scores based on positive ranks

^c^ *z*-scores based on negative ranks

At 18 months, all but two of the Q-CHAT items were significantly and positively associated with the total Q-CHAT score, with small to medium effect sizes (.12 ≤ *rho ≤* .47, all *p* < .001; items 5, *Protoimperative Pointing*, and 9, *Pretend Play*, *p* < .01; item 12, *Using hand as tool*, *p* < .05). Two items, *Echolalia* (item 18; *rho* = .01, *p =* .84) and *Looks at caregiver’s face to check reaction in unfamiliar situation* (item 21; *rho* = .10, *p =* .06) were not statistically significantly associated with the total score at 18 months.

At 24 months, all but two of the items significantly correlated with the total Q-CHAT score with small to large effect sizes (.15 ≤ *rho* ≤ .56; all *p* < .001; items 19, *Gestures*, and 21, *Looks at caregiver’s face to check reaction when faced with unfamiliar situation*, *p* < .01). Only *Echolalia* (item 18) and *Pretend play* (item 9) did not correlate significantly with the total Q-CHAT score (*rho* = −.04 and .10, respectively; both *p* > .05).

### Exploratory factor analysis of the Q-CHAT at 18 months

No previous factor analysis of the Q-CHAT has yet been published in the literature, with the exception of the unpublished factor analysis by Allison and colleagues [33]. For this reason, an EFA was carried out. The Kaiser-Meyer-Olkin (KMO) statistic was .79 (above the threshold of .50) and the Bartlett’s Test of Sphericity was significant (*χ*^2^ (300) = 1802.86, *p* < .0001), indicating that the Q-CHAT data at 18 months were suitable for EFA [44].

**Fig. 3:**
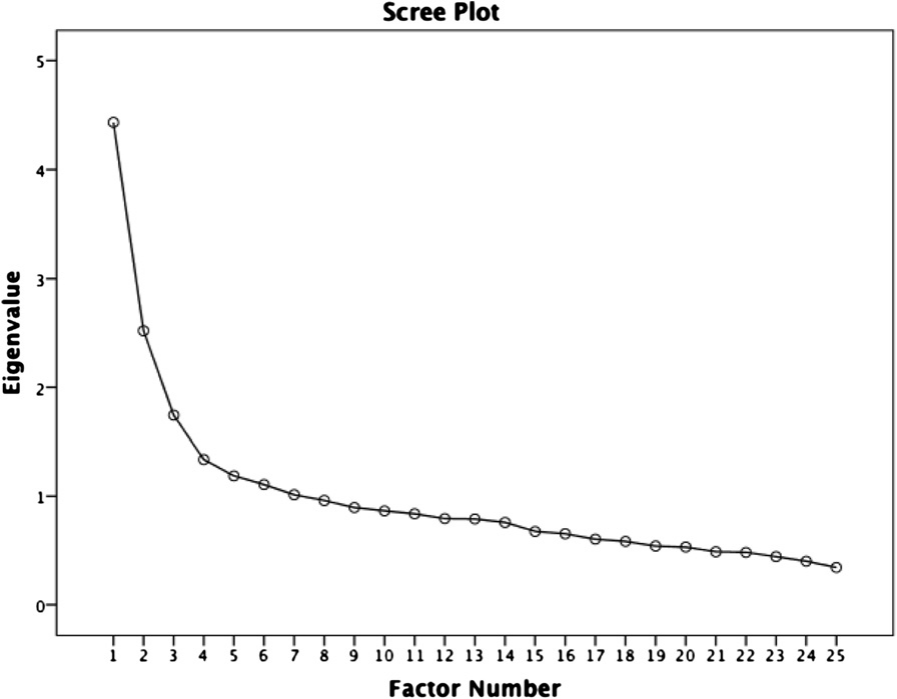
Scree plot of eigenvalues derived from exploratory factor analysis of the correlation matrix for 25 items of the 18-month Q-CHAT

Use of the Kaiser criteria initially suggested a solution of seven factors with Eigenvalues > 1, which explained 53.4 % of the total variance. However, examination of the scree plot (Fig. 3) indicated one point of inflexion after the third factor, thus the more parsimonious three-factor structure was selected. The EFA was re-run specifying three factors. A factor loading of 0.3 was chosen as the threshold above which items would be retained in the factor structure. Items 3 (*Lines objects up*), 13 (*Walks on tiptoes*), and 14 (*Ease of adapting to changes in routine*) were thus removed, as their factor loadings failed to reach the accepted threshold value. The resultant three factors explained a total of 38.1 % of the variance (factor 1, 19.5 %; factor 2, 10.95 %; and factor 3, 7.66 %). The pattern matrix, which employed direct oblimin rotation, is shown in Table 5.

**Table 5:** Pattern matrix (direct oblimin rotation) displaying factor loadings of EFA of the 22 items^a^ of the 18-month Q-CHAT

	Factors		
	1	2	3
10. Follows a look	*0.647*	−0.005	−0.079
6. Protodeclarative pointing	*0.617*	−0.007	0.017
1. Looks when name called	*0.580*	0.137	0.034
19. Gestures	*0.527*	0.076	−0.111
21. Check reaction	*0.506*	0.259	−0.142
2. Eye contact	*0.459*	−0.145	0.006
9. Pretend play	*0.445*	−0.095	−0.134
5. Protoimperative pointing	*0.436*	−0.072	0.054
12. Use of hand as tool^b^	*−0.423*	0.241	−0.147
15. Offer comfort	*0.313*	−0.139	−0.210
23. Twiddle objects repetitively^b^	−0.007	*0.628*	0.043
20. Unusual finger movements^b^	−0.065	*0.540*	−0.013
25. Stare at nothing with no purpose^b^	0.142	*0.475*	0.016
24. Oversensitive to noise^b^	0.093	*0.453*	0.019
16. Do same thing over and over again^b^	−0.195	*0.452*	−0.072
11. Sniff/lick unusual objects^b^	−0.112	*0.419*	−0.118
7. Interest maintained by spinning object^b^	0.002	*0.347*	0.057
22. Maintenance of interest^b^	−0.087	*0.339*	0.183
8. Number of words^b^	−0.059	0.066	*−0.666*
18. Echolalia^b^	−0.176	0.179	*0.508*
17. Typicality of first words	−0.044	−0.025	*−0.495*
4. Understand child’s speech	0.217	−0.006	*−0.481*

Extraction Method: Principal Axis Factoring. Rotation Method: Oblimin with Kaiser Normalization. Rotation converged in six iterations. Italicized entries indicate which items load on which factor

^a^Items 3, 13, and 14 excluded due to loading <0.3

^b^Reverse-scored items

**Fig. 4:**
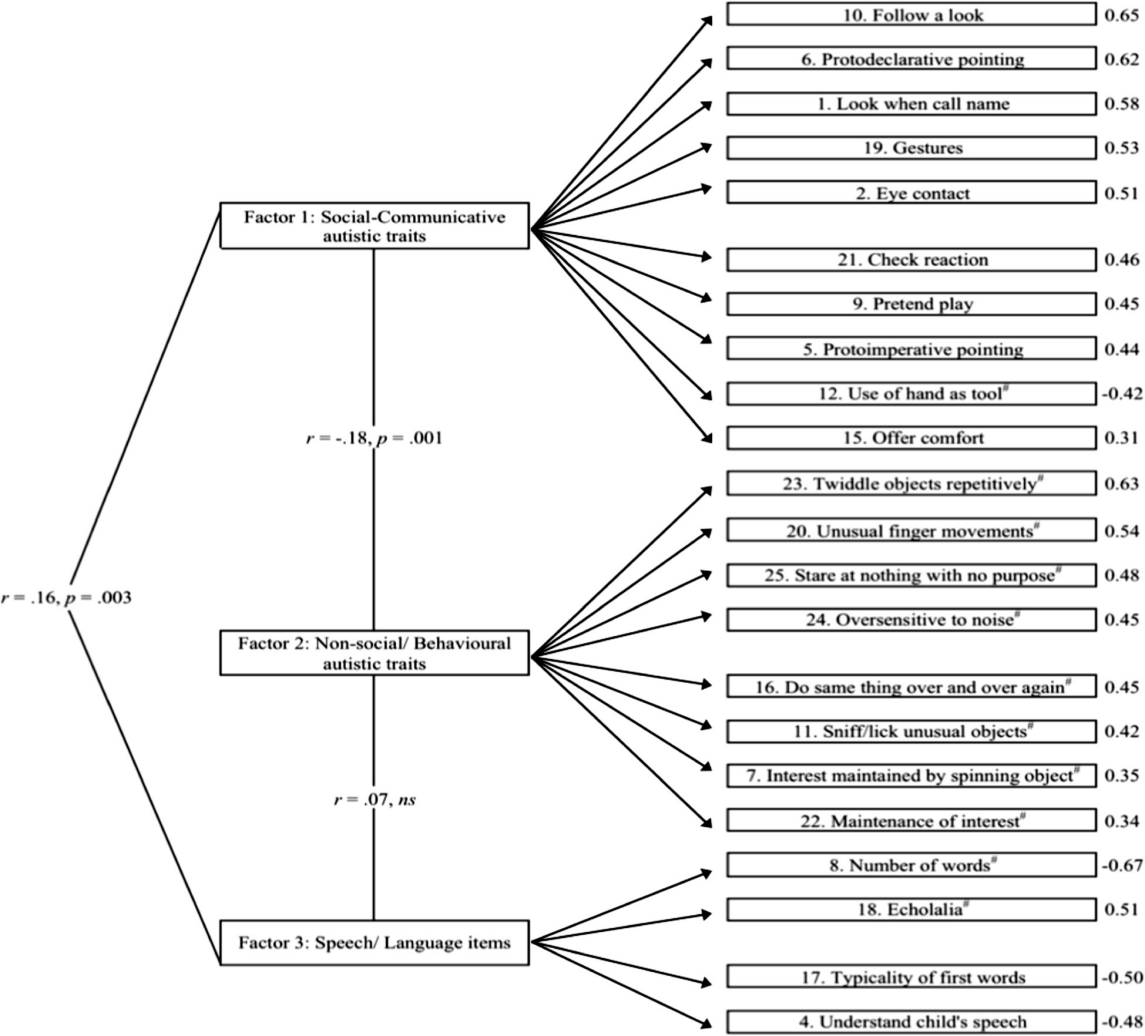
Three-factor structure of the Q-CHAT. Factor loadings beside individual items. *Number sign* reverse-scored items

Factor 1 (social-communicative autistic traits) comprised 10 items, of which only *Use of hand as tool* had a negative factor loading (item 12; the only reverse scored item in factor 1). Factor 2 (non-social/behavioral autistic traits) consisted of eight positively loaded items, all of which were reverse-scored. Finally, factor 3 (speech/language development) consisted of four items, of which only *Echolalia* (item 18) had a positive factor loading (see Fig. 4).

Following the factor analysis, raw factor scores were calculated for both time points through the summation of scores corresponding to items loading onto each factor (see Table 2 and Fig. 4*)*. Correlations between the three factor scores were of small effect sizes: *r* = −.18 and − .14 (factors 1 and 2 at 18 and 24 months, respectively; *p* = .001); .16 and .17 (factors 1 and 3 at 18 and 24 months; *p* = .003); and .07 and .19 (factors 2 and 3; *p* = .18, *ns* and < .01 at 18 and 24 months, respectively).

### Internal consistency

Internal consistencies of the Q-CHAT total and factor 3 (speech/language) items were suboptimal (*α* = .53 and .63 at 18 months; .60 and .63 at 24 months, respectively). Items in factors 1 (social-communicative autistic traits) and 2 (non-social/behavioral autistic traits) had acceptable internal consistencies (*α* = .76 and .69, respectively, at 18 months; .75 and .71 at 24 months). No item removal resulted in improved internal consistency values for any of the factor scores, except for the removal of item 18 (*Echolalia*), which somewhat improved the internal consistency of the total items from .53 to .57 at 18 months. However, the improved Cronbach’s alpha remained suboptimal.

### Stability and change in Q-CHAT total, factor, and item mean scores between 18 and 24 months

**Fig. 5:**
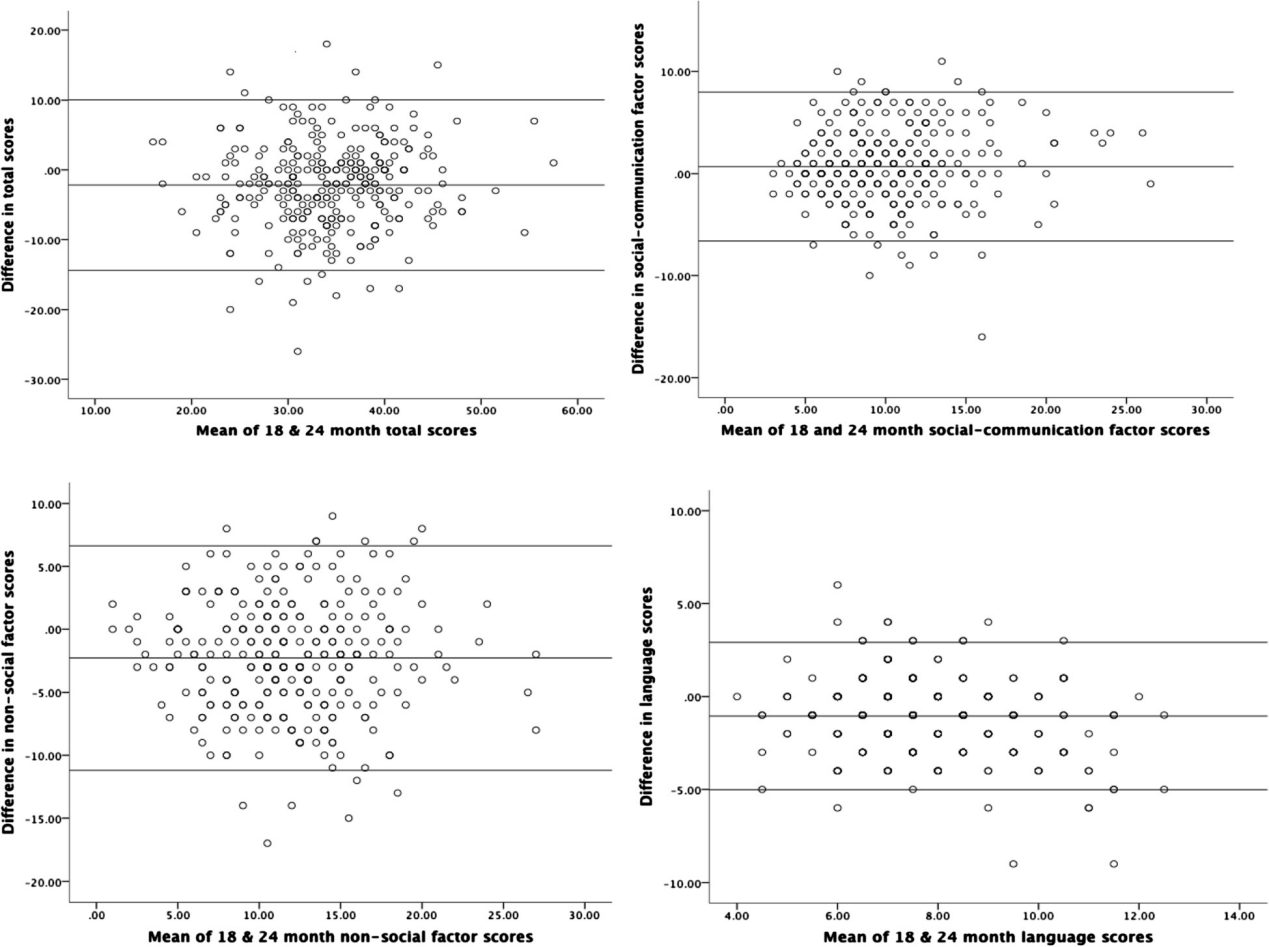
Bland-Altman plots for Q-CHAT differences between 18- and 24-month scores (total, social, non-social, speech/language factor scores)

The Q-CHAT total and factor scores at 18 months were highly positively correlated with the respective total and factor scores at 24 months with medium to large effect sizes (see Table 2). Most Q-CHAT individual items at 18 months also correlated significantly and positively with medium to large effect sizes with the same items at 24 months (.23 ≤ *r* ≤ .59; all *p* < .001; see Table 4). In addition to examining the relationship between 18- and 24-month Q-CHAT scores, the extent to which the scores obtained at the two time points were comparable (i.e., consistent) was also examined using Bland-Altman plots (see Fig. 5). Bland-Altman analysis is a graphical method which allows the comparison of two different measures (or two measurements obtained from the same measure at different time points). In this plot, the difference between each participant’s Q-CHAT 18- and 24-month score is plotted against the mean of the two measurements. The extent to which the two scores are comparable (in agreement) is defined by whether the difference scores largely fall within the two lines indicating the limits of agreement (+/−1.96 SD) [45]. Figure 5 shows that the Q-CHAT difference scores between 18 and 24 months are within two standard deviations of the average difference scores of the two measurements (lines of agreement), indicating overall stability and agreement (consistency) in autistic trait scores at the two time points.

Significantly fewer total autistic traits were reported by caregivers at 24 compared to 18 months with a medium effect size (see Table 2). Mean social-communicative autistic trait scores (factor 1) significantly increased from 18 to 24 months with a small effect size increase, while non-social/behavioral autistic trait scores (factor 2) decreased over time with a medium effect size (see Table 2). Finally, speech/language scores (factor 3) were significantly higher at 18 months as compared to 24 months with a large effect size, indicating, as developmentally expected, improvements in speech/language use (see Table 2).

At the item level, mean scores in items 4 (*Ease of understanding child’s speech*), 8 (*Number of spoken words*), 11 (*Sniffs/licks unusual objects*), 16 (*Does the same thing over and over again*), and 23 (*Twiddles objects repetitively*) decreased significantly from 18 to 24 months, with medium to large effect sizes, indicating lower frequency of these behaviors at 2 years of age. A significant mean score increase was observed from 18 to 24 months only for items 3 (*Lines objects up*) and 9 (*Engages in pretend play*), with medium effect sizes (see Table 4).

### Convergent validity of the Q-CHAT with the CBCL

Q-CHAT total scores at 18 and 24 months were significantly positively correlated with raw scores from the 24-month CBCL’s Internalizing Problems (*r* = .33 and .39, respectively), Withdrawn (.32 and .36), and PDD subscales (.30 and .35) with medium effect sizes (all *p* < .001). These three CBCL subscale scores were also significantly positively correlated with all three Q-CHAT Factor scores at both 18 and 24 months with small to medium effect sizes (.12 < *r < .*29, all *p* < .05), except factor 2 (non-social/behavioral items) at 18 months, which did not correlate significantly with the CBCL PDD scale at 24 months (*r* = .11, *p* = .07)*.*

## Discussion

This study aimed to utilize the Q-CHAT as a quantitative assessment of autistic traits in a non-clinical sample of Asian toddlers and to investigate and report its measurement properties and factor structure.

### Distribution of caregiver-reported autistic traits

The normal distribution of Q-CHAT scores at both 18 and 24 months is consistent with our present dimensional understanding of autistic traits. The higher mean autistic trait scores identified in this sample of Asian toddlers as compared to the original Q-CHAT study’s UK sample [17] are also in line with a small number of other studies also reporting higher autistic traits in Asian adults without ASD [11, 46]. In fact, our Q-CHAT data from unselected Singaporean toddlers with an average gestational age of 38.3 weeks at birth (SD = 1.5 weeks) were comparable to those reported by Wong and colleagues [30] in prematurely born children from the UK. In a recently published study of Chinese school-aged children without ASD, CAST mean scores (*M* = 7.8, SD = 3.7) [47] were also significantly higher than those reported in the original CAST prevalence study in the UK [26] with a large effect size difference (*M* = 4.73, SD = 3.57; calculated *d* = .84). However, not all studies have consistently reported cross-cultural differences in mean scores obtained from caregiver-reported measures of autistic traits (i.e., [19, 21]). Thus, it is possible that differences in cultural experiences and interpretation of childhood behaviors may only partly explain the observed differences in caregiver- or self-reported autistic traits between the two samples (see [48] for a review of autism across cultures). Our sample was also demographically different to that of Allison and colleagues [17], as mean age at which mothers left formal education was higher in our study, with a large calculated effect size difference (*d* = .64). Other studies have failed to find significant ethnic/country differences (i.e., [49, 50]) and, interestingly, the mean CBCL PDD subscale raw score in our sample (*M* = 3.71; SD = 3.21) was comparable to the “omnicultural” mean PDD subscale raw score reported in [37] from 24 societies, including Singapore. Thus, although it is possible that ethnic/cultural differences and family/child differences may affect caregiver report of children’s autistic traits, this has yet to be investigated systematically (see, however, [51] for recent work on this) and further research is required to better understand possible socio-cultural influences in caregiver- or self-reported autistic traits.

### Q-CHAT factor structure

Our study proposed a three-factor structure of Q-CHAT autistic traits in an unselected sample: a social-communicative factor, a non-social/behavioral factor and a speech/language factor. These were the same three factors that were identified in the unpublished factor analysis of the Q-CHAT [33]. Although derived in an unselected sample, the first two factors—social/communicative and non-social/behavioral autistic traits—reflect the DSM-5’s dyad of diagnostic criteria for ASD [1], providing some evidence towards a similar factor structure of autistic traits and related symptoms across both unselected and clinical populations. Other recent factor analytic studies in unselected and clinical samples also support the clustering of social communication and interaction items in a single factor (e.g., [52, 53]) and the independent contribution of stereotyped and repetitive behaviors as a separate factor [54, 55]. Finally, the third Q-CHAT factor, comprising only four items, is not specific to autistic traits, but is more developmental in its focus on speech/language.

Other studies examining other measures of autistic traits or symptoms of ASD have proposed similar factor structures to the one found in the present study for the Q-CHAT. Sun and colleagues [47] recently examined the psychometric properties of the Mandarin Chinese version of the CAST [26] in 737 4–11-year-old children from Mainland China. They found a similar symptom presentation to that reported in Western populations and a two-factor solution for its 28 items (social/communication factor and stereotyped/inflexible language and behavior factor). Matson et al. [56] also showed a three-factor structure of socialization/non-verbal communication, repetitive behavior/restricted interests, and communication on the Baby and Infant Screen for Children with aUtIsm Traits—Part 1 (BISCUIT—Part 1).

### Measurement properties of the Q-CHAT

Internal consistencies were suboptimal for the Q-CHAT total and Q-CHAT speech/language factor scores (ranging from .52 to .63), but acceptable for the two main Q-CHAT factors of social/communication and non-social/behavioral autistic traits (.69 to .76). Rather than being a unitary measure, as suggested by researchers employing other measures for which a single factor structure has been proposed (e.g., [52, 57]), our findings suggest that autistic traits may be better conceptualized as social and non-social traits. The small correlation between these two factor scores (*r* = −.18) highlights their largely independent relationship (see also [54, 58, 59] for a research review). Test-retest reliability was .60–.64 for the total and the two main factor scores after 6 months, and the Bland-Altman analyses showed overall consistency in the scores from 18 to 24 months. Higher test-retest reliability of .82 for the Q-CHAT was reported by Allison et al. [17], but re-test in their study was only 1 month later; likewise, higher test-retest reliabilities have been reported for the SRS [6]; however, most other studies either investigated autistic traits in older children, where such traits are likely to be more stable, or carried out the second assessment a few weeks only after the first one, rather than after a longer interval of 6 months, as in the present study.

Finally, examination of convergent validity of the Q-CHAT total and factor scores showed that all scores, except factor 3 speech/language scores, were positively associated with the CBCL’s PDD subscale with a medium effect size. Autistic-trait-related behaviors at 18 and 24 months were also positively associated with internalizing and withdrawn behaviors at 24 months and autistic symptoms as measured by the CBCL, providing evidence that the relationship between internalizing traits and autistic traits found in school-aged children [60] is also present in unselected samples of toddlers as young as 18 months. Constantino et al. [52] and Duku et al. [61] reported similarly positive, but larger, correlations between the SRS and CBCL internalizing scores (.47 and .68) in their studies of 4- and 9-year-old children with diagnoses of ASD, respectively.

### Stability and change in Q-CHAT scores from 18 to 24 months

Overall, Q-CHAT total and factor scores were generally stable between 18 and 24 months. Individual Q-CHAT items also showed high mean stability after 6 months. Nevertheless, there was also evidence of change over time: mean non-social/behavioral autistic factor scores decreased significantly (indicating fewer autistic traits) during this period (*d* = .45), as did mean speech/language development factor scores (*d* = .56). These changes in factor and item scores are likely expected as part of normative development in young children. For example, there was a significant reduced frequency of repetitive action (*item 16*, *d* = .51) and significantly increased number of spoken words (*item 8*; *d* = .72) by 24 months. These changes are indicative, respectively, of the decrease in obsessionality and levels of high repetition (i.e., [62]) and the improvements in language comprehension and verbal expression of typically developing toddlers. This trajectory of decreasing repetitive speech and behavioral patterns in unselected toddlers likely contrasts a trajectory of persisting or increasing such behaviors in children with ASD (i.e., [63, 64]).

### Limitations and future directions

This study is one of the first to quantify the distribution of autistic traits in an unselected population of young Asian toddlers. The organization of autistic traits into social and non-social/behavioral traits proposed by this study based on EFA is consistent with the DSM-5’s current structure for ASD. This was a prospective study, thus avoiding recall bias from caregiver reports [65].

As this study was embedded in the larger multi-time point GUSTO study, some data were lost due to participant attrition or non-participation at certain time points, resulting in a lower sample size available for analyses when making comparisons between measures or examining the Q-CHAT over the two time points. Return rates for the Q-CHAT were thus modest. However, Q-CHAT respondents’ characteristics did not differ significantly from the main GUSTO cohort, making our results likely generalizable to the larger cohort. In addition, internal consistencies of the total Q-CHAT score and the four-item speech/language factor score were suboptimal. Finally, there is no certainty if the same informant provided information about the children’s autistic traits and emotional/behavioral problems at 18 and 24 months in the Q-CHAT and CBCL, as information about the identity of the informants was not collected at 18 months. However, the majority of respondents in the GUSTO study were mothers across all measures and time points.

## Conclusions

To summarize, this study showed autistic traits that are continuously distributed in an unselected sample of Asian toddlers and proposed a three-factor structure for the Q-CHAT with two autism-specific factors organized into social and non-social/behavioral autistic traits. Q-CHAT scores after 6 months were generally stable, with repetitive behaviors showing a developmentally expected decrease. There was evidence of convergent validity with the CBCL-PPD subscale and acceptable internal consistency for the two main factor items, but internal consistency was suboptimal for the Q-CHAT total and the speech/language factor items.

Future work should aim to validate this study’s factor structure with other diverse unselected samples as well as with children diagnosed with ASD before the age of 2½–3 years. Studying continuity and change in autistic traits over the toddler and pre-school years and into childhood would also strengthen our understanding of the trajectory of autistic traits over time in unselected children and would allow more fruitful and informative comparisons with early developmental trajectories of children with ASD in the first 2 years of life. As most of the measures currently developed to assess autistic traits rely on informants, attention should also be paid in exploring the potential influence of child characteristics or informant factors (i.e., caregiver stress or depression, parental education, etc.) in the interpretation and rating of items examining autistic traits.

### Endnotes

^1^Between June 2009 and September 2010, pregnant mothers over 18 years old were recruited from the two largest public birthing hospitals in the country. They were Singaporean Citizens or Permanent Residents intending to live in Singapore for the next 5 years and of Chinese, Malay, or Indian ethnicity with homogeneous parental ethnic background. Mothers who miscarried, received chemotherapy or psychotropic drugs during pregnancy or had pregnancy complications (i.e., pre-eclampsia, gestational diabetes) were excluded.

^2^In recognition of the constraints of conducting such detailed assessments on the full GUSTO sample and in line with the main research aims of the other GUSTO investigators, priority for inclusion in the GUSTO neurodevelopmental cohort was given to infants who had participated in previous neurodevelopmental assessments in the first weeks of life, ethnic minorities (Malays and Indians), infants who had high or low intakes of breast milk and participants who through word of mouth or interest contacted the neurocognitive team and volunteered for more detailed neuropsychological assessment.

^3^Detailed data on the relationship of the Q-CHAT respondent with the child were not collected at 18 months, but the majority of respondents (>80 %) were mothers across all caregiver-reported measures in the GUSTO study.

## Abbreviations

ASD: autism spectrum disorder
ASSQ: Autism Spectrum Screening Questionnaire
AQ: Autism Spectrum Quotient
BAP: broader autism phenotype
BISCUIT: Baby and Infant Screen for Children with aUtIsm Traits
CAST: Childhood Autism Screening Test
CBCL: Child Behavior Checklist
GUSTO: Growing Up in Singapore Towards Healthy Outcomes
EFA: Exploratory Factor Analysis
SRS: Social Responsiveness Scale
Q-CHAT: Quantitative Checklist for Autism in Toddlers
